# Boosting the Sensitivity and Hysteresis of a Gel Polymer Electrolyte by Embedding SiO_2_ Nanoparticles and PVP for Humidity Applications

**DOI:** 10.3390/gels10010050

**Published:** 2024-01-10

**Authors:** Michelle Cedeño Mata, Albert Orpella, Manuel Dominguez-Pumar, Sandra Bermejo

**Affiliations:** MNT Group, Electronic Engineering Department, Polytechnic University of Catalonia (UPC), C/Jordi Girona 1-3, 08034 Barcelona, Spain

**Keywords:** gel polymer electrolytes, ionic liquid, colloidal nanoparticles, impedance spectroscopy

## Abstract

Enhancing sensitivity and hysteresis in capacitance humidity sensors is vital for precise, reliable, and consistent humidity control. This study explores this concern by incorporating polyvinylpyrrolidone (PVP) and SiO_2_ nanoparticles into a polyvinyl alcohol (PVA)-based ionic liquid gel polymer electrolyte (ILGPE), studying two capacitor types: ILGPE and SiO_2_ composite ILGPE (CILGPE) capacitors. These novel electrolytes use ammonium acetate as a plasticiser, 1-butyl-3-methylimidazolium bromide as an ionic liquid, SiO_2_ nanoparticles as a composite, and PVA and PVP as host polymers. Capacitors were characterised and modelled using impedance spectroscopy (IS), providing an electrophysical insight into their working principle. Sensitivity and hysteresis were evaluated within a 20–90% relative humidity (RH) range at 25 °C. The SiO_2_ CILGPE capacitor with PVP presented superior sensitivity and hysteresis, revealing the beneficial combination of SiO_2_ nanoparticles and PVP. These benefits are due to the creation of pathways that facilitate water molecule diffusion and crystallinity reduction in PVA-ILGPE. In particular, at 10 kHz, it demonstrates a calibrated capacitance sensitivity of 2660 pF/%RH and a hysteresis of 3.28 %RH. This optimised capacitor outperforms some previous humidity capacitive sensors in sensitivity while exhibiting low hysteresis.

## 1. Introduction

Accurate control and monitoring of environmental conditions are vital in various domains, including agriculture, healthcare, and industrial processes. Therefore, a lot of research has been focused on developing sensors that exhibit high sensitivity, low hysteresis, and rapid linear response and recovery over a wide range of humidity without compromising the cost [1]. Several studies have highlighted sensors capable of detecting humidity changes through detecting alterations in parameters such as impedance, voltage, capacitance, resistance, or frequency. Among these, impedimetric and capacitive sensors stand out due to their simple and cost-effective manufacturing advantages, easy use and integration, low power consumption, and good performance. Moreover, capacitive devices have been in the limelight of the sensing field due to their long operating life and design flexibility [2]. These factors, coupled with the ease of capacitance amplification via oscillator circuits, have propelled these devices to a dominant position in the market [3].

Particularly in the sensing field, the study of sensitive materials has been considered an important research topic due to the wide range of suitable materials and combinations that lead to an easy driving of the humidity-sensing performance and mechanism. In this context, polymers, electrolytes, ceramics, and carbon-based materials have become common moisture-sensing materials [1,4]. Among these humidity-sensitive materials, polymers have been proven to be excellent materials for humidity sensing due to their compatibility with other sensing materials and sensitivity to %RH variation, mainly exhibited in their dielectric constant and conductivity. Furthermore, polymers offer benefits such as low cost, low weight, resistance to corrosion, easy and flexible fabrication, high stability, and commercial potential, which make them suitable materials for humidity-sensing applications. Polymers used for humidity sensors include polyvinyl alcohol (PVA) [5,6], polyvinylpyrrolidone (PVP) [7,8], polypyrrole (PPy) [9], keratin [10], and Nafion [11]. Among these polymers, PVA has been widely used due to its properties, such as non-toxicity, biocompatibility, highly hygroscopic nature, and hydrophilicity [5,6].

Nevertheless, PVA-based humidity sensors often have low sensitivity and slow transient responses to relative humidity variations, mainly attributed to PVA’s low ion conductivity and high intrinsic impedance. Therefore, several studies combine PVA with other materials to address these limitations and enhance humidity-sensing performance [5]. The electrical and humidity-sensing performance of PVA-based films can be improved by incorporating polymers, plasticisers, ionic liquids (ILs), and doping fillers.

In particular, incorporating ceramic nanostructured materials into PVA-based humidity-sensing layers has revealed significant improvements. Hydrophilic ceramics exhibiting excellent thermal and chemical stability are well suited as humidity-sensitive materials. The effectiveness of these nanostructures in humidity sensing can mainly be attributed to their large surface area, which offers numerous sites for water interaction. Oxide ceramics such as titanium oxide (TiO_2_) [12,13], alumina (Al_2_O_3_) [14], barium titanate (BaTiO_3_) [15], and silicon oxide (SiO_2_) [16] demonstrate strong responses to humidity changes due to their excellent water adsorption and desorption properties. Additionally, clay ceramics like palygorskite [17], sepiolite [18,19], and halloysite [20] also attract attention for their cost-effectiveness and environmentally friendly nature. Halloysite nanotubes (HNTs) are widely used due to their excellent biocompatibility. However, their brittleness and heterogeneity in size, surface charge, and formation of hydrogen bonds require additional surface modification processes to overcome these challenges and enhance their performance [21,22]. These concerns, in combination with the maturity of oxide ceramic surface-treatment techniques, their excellent properties, and their reliable long-term performance, make oxide ceramics more suitable for humidity sensing and as effective fillers for PVA-based humidity sensors in terms of stability and durability.

On the other hand, regarding ILs, their integration into polymers leads to flexible materials that possess high ionic conductivity and good mechanical properties and stability. ILs have been recognised as green solvents that serve as excellent plasticisers for polymers [23,24,25]. Their tunability, thermal stability, intrinsic ionic conduction, nonflammability, and low volatility make them highly effective for sensing applications [23,24,26,27], either as modifier materials for electrodes or as sensitive materials [23,25]. In particular, combining hygroscopic ILs and polymers in humidity sensors leads to materials with enhanced mechanical properties, improved ionic conductivity, and boosted humidity-sensing capabilities. Among different ILs, the 1-butyl-3-methylimidazolium (Bmlm) class, in combination with polymers such as polyvinylidene fluoride (PVDF) and PVA, has shown promising results. For instance, in [25], a highly sensitive humidity sensor based on 1-butyl-3-methylimidazolium tetrachloroferrate (BmlmFeCl_4_) and PVDF is exhibited. In contrast, 1-butyl-3-methylimidazolium bromide (BmlmBr) is a hygroscopic, environmentally friendly, low-cost IL. Its combination with PVA has been widely studied, demonstrating its crucial beneficial role as a plasticiser [28].

Finally, the strategic approach of combining different polymers to improve electrical and humidity-sensing performance is rooted in the leveraging of the strengths of each polymer and their mutual tunability. In humidity sensors, amalgamating polymers with different affinities for water molecules or porous or amorphous structures can lead to a sensing layer with fine-tuned humidity-sensing performance. In particular, some works exhibit the combination of PVA/PVP sensing films as a good alternative for humidity sensors [29,30]. PVP is an amorphous polymer that exhibits high compatibility with PVA. PVP can improve PVA’s physical properties by reducing its crystallinity and protecting the electrolyte during drying, preserving its structural integrity [29,30].

This work presents the synthesis and characterisation of ionic liquid gel polymer electrolytes (ILGPEs) formulated using PVA, PVP, ammonium acetate, and 1-butyl-3-methylimidazolium bromide. Additionally, composite ILGPEs (CILGPEs) have been developed by embedding SiO_2_ nanoparticles that act as fillers. PVA has been selected as the host polymer because of its chemical stability, capacity to absorb water, and non-toxicity. PVP has been included as a lyophilised agent that can also boost the humidity-sensing performance. Ammonium acetate has been selected as a plasticiser due to its effective plasticising properties, while 1-butyl-3-methylimidazolium bromide, an ionic liquid, has been blended in to enhance ionic conductivity. Finally, nonporous silica nanobeads have been selected as a composite material due to their high stability, biocompatibility, hydrophilicity, and high surface area, all of which are promising properties for humidity sensing. In previous research, Véliz et al. demonstrated the effectiveness of nonporous SiO_2_ nanoparticles in sensing humidity changes [31]. Moreover, Cedeño et al. revealed the constructive impact of incorporating silica nanofillers into ILGPE [32]. In particular, SiO_2_ nanoparticles promote structural changes by forming amorphous regions and incorporating additional pathways for ion transportation. Such findings could be beneficial for humidity sensitivity and hysteresis, leading SiO_2_ nanofillers to be suitable materials for humidity sensors. By incorporating and combining PVP and SiO_2_ nanoparticles, the humidity-sensing performance of the ILGPE- and SiO_2_ CILGPE-based capacitors has been driven.

Consequently, this research compares the impedance properties of different ionic liquid gel polymer electrolyte-based capacitors, delving into the subtle effects of their composition. It investigates the influence of PVP and SiO_2_ nanoparticles on the humidity sensitivity and hysteresis of a PVA-based ILGPE capacitor across a relative humidity (RH) range of 20 to 90%. This work intends to provide an initial insight into the potential of these ILGPEs and SiO_2_ CILGPEs as humidity-sensing layers. Furthermore, by studying the effect of PVP and silica nanofillers on sensitivity and hysteresis, this work aims to identify an optimal electrolyte composition regarding these critical parameters. The novelty of this work lies in three points: (i) the amalgamation of various strategies for tuning and boosting the humidity-sensing performance of a PVA-based ILGPE, (ii) the novel approach of combining PVP and SiO_2_ nanoparticles with a PVA-based ILGPE for humidity sensing, and (iii) the introduction of novel formulated ILGPEs that incorporate SiO_2_ and PVP, presenting an unexplored composition with optimised humidity-sensing capabilities.

## 2. Results and Discussion

### 2.1. Impedance Spectroscopy (IS) Characterisation and Modelling

**Figure 1:**
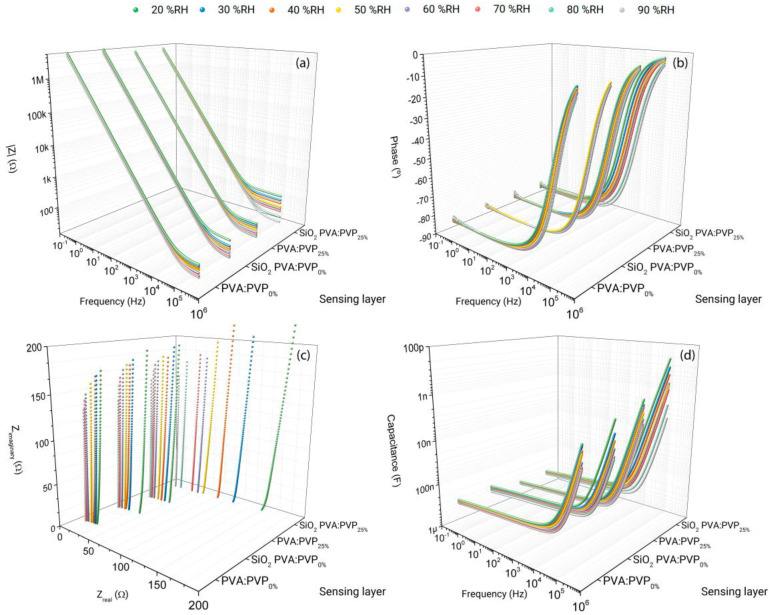
Impedance data comparison of PVA:PVP_0%_-ILGPE-, SiO_2_ PVA:PVP_0%_-CILGPE-, PVA:PVP_25%_-ILGPE-, and SiO_2_ PVA:PVP_25%_-CILGPE-based capacitors. (**a**) Impedance module vs. frequency vs. electrolyte content; (**b**) phase responses vs. frequency vs. electrolyte content; (**c**) Nyquist vs. electrolyte content; and (**d**) capacitance vs. frequency vs. electrolyte content. *|Z|*, *Z″*, and *Z*′ represent the impedance modulus and the imaginary and real parts of the complex impedance, respectively.

The IS method has been employed to investigate the electrical behaviour of the fabricated devices. Capacitance and equivalent series resistance (ESR) have been obtained and analysed to assess the impact of the employed materials and their concentrations on the electrical and humidity-sensing performance. Figure 1a,b illustrate the obtained bode plots of the fabricated devices at different relative humidity levels during the adsorption process. Figure 1c,d compare the Nyquist and capacitance plots, respectively. In particular, capacitance (*C′*) curves have been calculated from the following equation:

(1) $$C^{\prime }=\frac{Z^{\prime \prime }}{2\pi f{\left|Z\right|}^{2}}$$

where *|Z|* is the modulus and *Z″* is the imaginary part of the complex impedance.

Figure 1 depicts the impedance spectrum data obtained from PVA:PVP_0%_, SiO_2_ PVA:PVP_0%_, PVA:PVP_25%_, and SiO_2_ PVA:PVP_25%_ capacitors at various relative humidity (RH) levels, thereby exhibiting the trend of the electrical behaviour as a function of %RH and sensing layer composition. Figure 1a compares the impedance modulus of the fabricated devices at different relative humidity levels. As can be noticed, all the capacitors exhibit a similar impedance modulus behaviour, thereby revealing two distinct trends. The first trend is characterised by a drop in the impedance modulus, with a slope of −1 from 0.1 Hz to a specific frequency, indicative of a capacitive behaviour. After this particular frequency, the impedance modulus flattens, exhibiting a slope equal to 0 and tending towards a particular value, revealing a transition from a capacitive to a resistive behaviour. Therefore, the frequency at which the impedance modulus changes its slope acts as a threshold frequency. This behaviour change can also be observed in Figure 1b, where, between a frequency of 0.1 Hz and the specific threshold frequencies, the fabricated devices show phase values smaller than −45°, which are attributed to a capacitive behaviour. Beyond these threshold frequencies, the phase responses increase, with their values approaching 0°, indicative that at high frequencies, resistive behaviour predominates. The second trend observed relies on the variation in the impedance modulus in response to changes in humidity levels. This trend is characterised by a reduction in the impedance modulus values with increasing relative humidity. This finding is also evident in Figure 1c, where the Nyquist plots intersect with the real impedance axis at lower values. This phenomenon can be attributed to an enhancement of the charge contribution due to increased charge carrier density. A higher density of charge carriers may result in increased capacitance values. Consequently, the capacitance spectres also reveal increased values in response to a humidity increase (Figure 1d). This behaviour is widely exhibited by capacitive humidity sensors [16,33,34].

**Figure 2:**
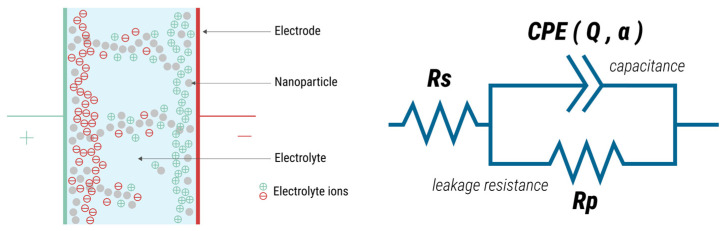
Schematic representation of the SiO_2_ CILGPE-based capacitor and its equivalent circuit. *R_s_*, *R_p_*, and *CPE* represent the series resistance, the leakage resistance, and the constant phase element, respectively.

The overall trend exhibited in Figure 1 can be fitted according to the Cole–Cole model as an equivalent circuit (Figure 2). In particular, Figure 1c compares the obtained Nyquist plots, wherein part of a characteristic semi-circular shape is exhibited by these curves, revealing a capacitor and resistor connected in parallel. The semi-circular shape represents the capacitive behaviour of the fabricated composite and non-composite ILGPE-based capacitors. Nevertheless, at high frequencies, the semi-circular responses present some shifts from the origin, indicating the existence of a resistance in series with the parallel combination. This deviation is typically ascribed to the bulk resistance of the materials that comprise the capacitors, demonstrating that two sub-circuits connected in series form the equivalent circuit.

The first sub-circuit within the equivalent circuit model comprises a series resistor (*R_s_*). Meanwhile, the other circuit involves the parallel combination of a constant phase element (*CPE*) and a resistor (*R_p_*) related to a leakage resistance. The CPE is a frequency-dependent element that simulates the formation of a double-layer capacitance. The *CPE* appears due to the charge accumulation at the electrode–electrolyte interfaces, as well as at the boundaries of the nanoparticles. This element can be defined by Equation (2).

(2) $$Z_{CPE}=\frac{1}{Q_{CPE}{\left(j2\pi f\right)}^{\alpha }}$$

*Q_CPE_* represents the admittance of an ideal capacitance, and α is the parameter employed to model the frequency dispersion resulting from non-ideal capacitive behaviour owing to factors such as electrode roughness or electrolyte inhomogeneity [35,36].

The *R_s_* represents the ESR, which describes the combination of the internal resistive effects of the materials that compose a capacitor. Therefore, *R_s_* arises from the bulk resistance of the electrolyte and the electrodes internal resistance [36]. Specific frequency ranges have been selected to illustrate a portion of the Nyquist plots (Figure 1c) to appreciate each device’s exhibited ESR values at different %RH levels. The *R_s_* values have been estimated by determining the intersections of the Nyquist curves with the x-axis at higher frequencies [35,36].

### 2.2. Surface Topography

**Figure 3:**
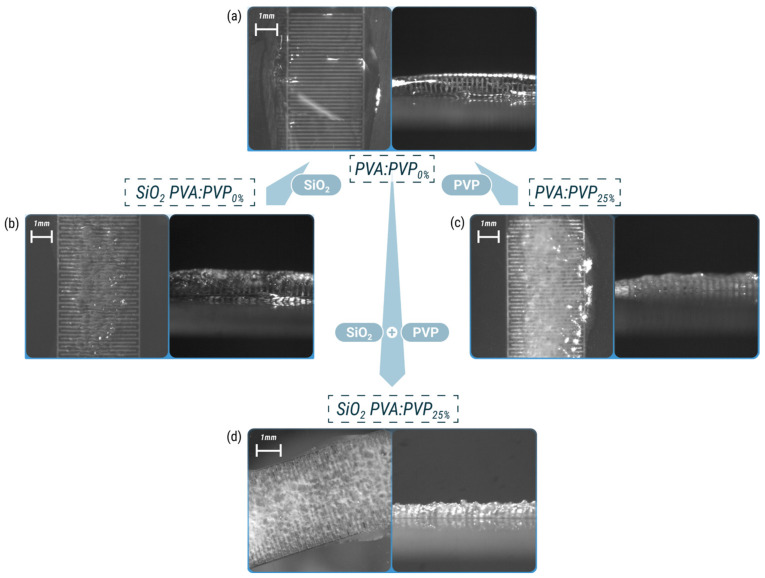
Plan and side views of a (**a**) PVA:PVP_0%_-ILGPE-, (**b**) SiO_2_ PVA:PVP_0%_-CILGPE-, (**c**) PVA:PVP_25%_-ILGPE-, and (**d**) SiO_2_ PVA:PVP_25%_-CILGPE-based capacitors.

Figure 3 illustrates both plan and side views of the PVA:PVP_0%_, SiO_2_ PVA:PVP_0%_, PVA:PVP_25%_, and SiO_2_ PVA:PVP_25%_ capacitors. In particular, Figure 3a shows the top surface and side profile of the PVA:PVP_0%_-based capacitor. As expected, the PVA:PVP_0%_-ILGPE composite exhibits a transparent and smooth surface topography that reflects its homogeneity and uniformity.

Incorporating silica nanoparticles or PVP into PVA:PVP_0%_-ILGPE stimulates topography changes. SiO_2_ PVA:PVP_0%_- and PVA:PVP_25%_-based electrolytes reveal slight roughness regions. These textured regions suggest a degree of amorphousness within the layers, ascribed to the capability of PVP and SiO_2_ nanofillers to disrupt the crystalline structure of PVA. The influence of PVP in diminishing the crystal phase of PVA is also evident in [37,38,39]. The emergence of this topography transformation leads to an increase in the air-contact area of the sensing layer, which could positively affect the humidity-sensing performance.

The sensing layer integrating SiO_2_ nanoparticles and PVP into PVA:PVP_0%_-ILGPE shows a distinct topography. The SiO_2_ PVA:PVP_25%_-CILGPE exhibits a translucent quality and presents a more pronounced surface roughness than the PVA:PVP_0%_-, SiO_2_ PVA:PVP_0%_-, and PVA:PVP_25%_ -based ILGPEs. This effect could be due to the simultaneous presence of PVP and silica fillers, which increases the concentration of agents disrupting the crystalline phase of PVA. The appearance of a rougher topography implies an increase in the air-contact surface area of the sensing layer, suggesting the enhancement of the sensitivities exhibited by SiO_2_ PVA:PVP_0%_- and PVA:PVP_25%_-based capacitors.

### 2.3. Humidity Sensor Performance

**Figure 4:**
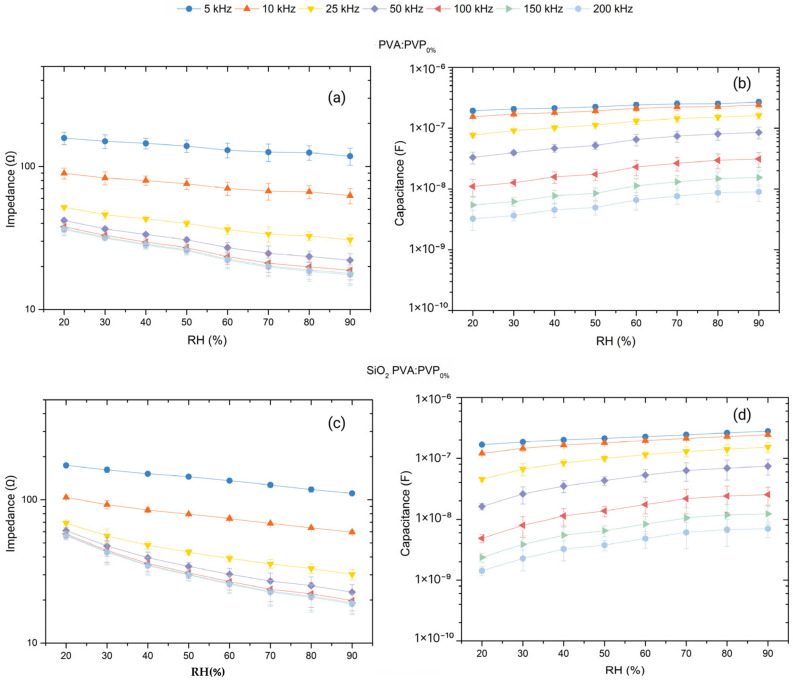

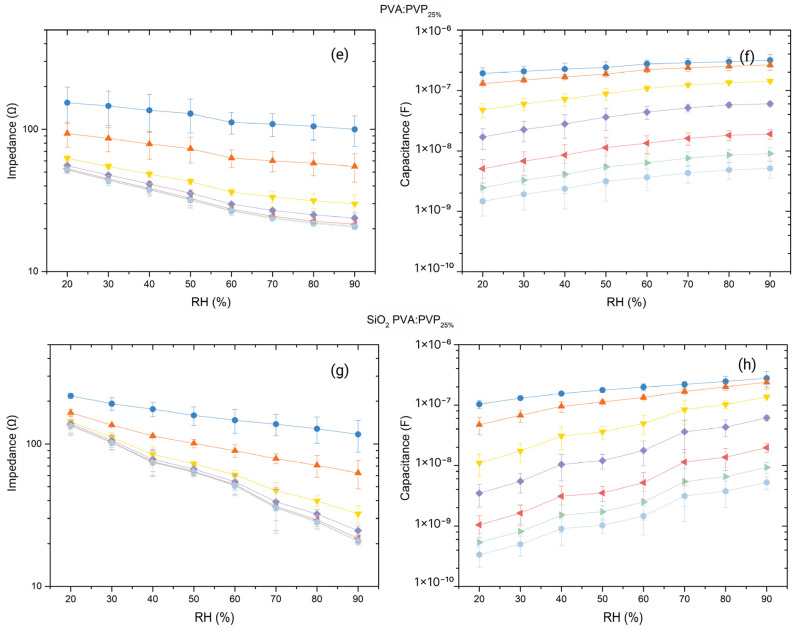
Impedance modulus and capacitance humidity-sensing responses at the evaluated frequencies exhibited by (**a**,**b**) PVA:PVP_0%_-ILGPE-, (**c**,**d**) SiO_2_ PVA:PVP_0%_-CILGPE-, (**e**,**f**) PVA:PVP_25%_-ILGPE-, and (**g**,**h**) SiO_2_ PVA:PVP_25%_-CILGPE-based capacitors.

The humidity impedimetric and capacitive responses of the different fabricated ILGPE- and SiO_2_ CILGPE-based capacitors have been evaluated at various frequencies in a relative humidity range from 20 %RH to 90 %RH. Seven different %RH levels have been applied to test the impedance and capacitance responses during the adsorption and desorption processes. The responses of three PVA:PVP_0%_, SiO_2_ PVA:PVP_0%_, PVA:PVP_25%_, and SiO_2_ PVA:PVP_25%_ capacitors are displayed in Figure 4.

As illustrated in Figure 4, the impedance responses of the fabricated humidity sensors exhibit a decreasing trend with relative humidity; meanwhile, the capacitance responses reveal an increasing trend due to the relative humidity increase. The exhibited impedance and capacitance behaviours could be ascribed to the enhancement of ion conduction resulting from the interaction of the sensing layer with water molecules. Higher relative humidity levels imply an increase in the probability of the adsorption of water molecules and, hence, an increase in the charge carrier density. As a result, the developed capacitors’ impedance diminishes, and capacitance rises. Regarding the role of the operating frequency, it can be noticed that the impedance and capacitance responses tend to exhibit lower variations at high frequencies, indicating a decrease in the sensitivity of the ILGPE and SiO_2_ CILGPE capacitors with the frequency. This effect can also be ascribed to water molecules’ polarisation dynamics. Adsorbed water molecules can follow changes in the electric field at low frequencies. However, they are not able to follow faster electric field changes that occur at high frequencies [10,16].

Furthermore, it is noticeable that the PVA:PVP_0%_ capacitors reveal linear impedance responses to humidity changes. However, adding SiO_2_ nanoparticles, PVP, or both stimulates the interaction with water molecules, leading to impedance responses displaying non-linear behaviours. This observation reduces the viability of using impedance modulus as a humidity-sensing parameter since one of the desired sensor characteristics is linearity. Consequently, evaluations of sensitivity and hysteresis have been exclusively focused on the capacitance responses.

**Figure 5:**
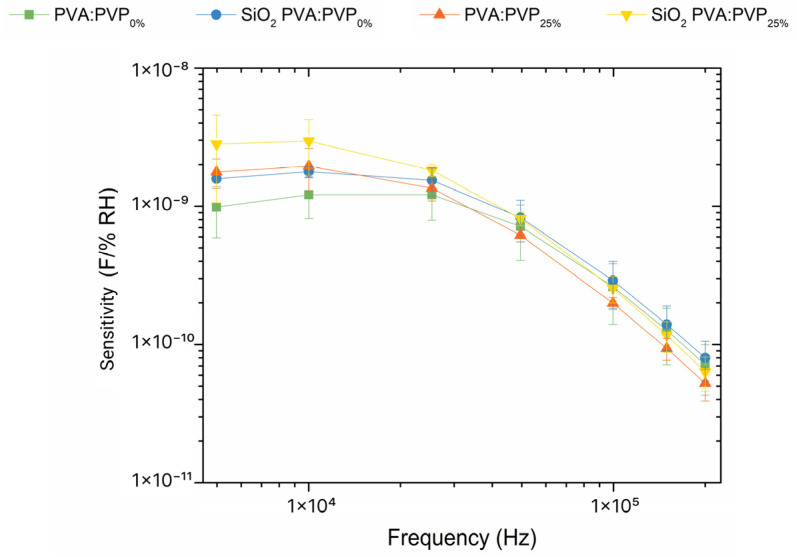
Comparison of the capacitance sensitivities exhibited by PVA:PVP_0%_-ILGPE-, SiO_2_ PVA:PVP_0%_-CILGPE-, PVA:PVP_25%_-ILGPE-, and SiO_2_ PVA:PVP_25%_-CILGPE-based capacitors at the evaluated frequencies.

Following the analysis of the exhibited humidity-sensing responses at different frequencies, the capacitance sensitivities of the sensors to changes in relative humidity in the ambient chamber at 25 °C have been calculated using Equation (3), as typically used [1,13,34,40,41]:

(3) $$S_{\gamma }=\frac{\Delta_{y}}{\Delta_{RH}}=\frac{\gamma_{90\%}-\gamma_{20\%}}{90-20}$$

where, $\gamma_{90\%}$ and $\gamma_{20\%}$ are the capacitance values at 90% and 20% of relative humidity, respectively. Figure 5 compares the sensitivity values vs. the evaluated frequencies and shows an evident impact on capacitance sensitivities because of the addition of PVP and SiO_2_ nanofillers to the PVA-based ILGPE. The exhibited capacitance responses indicate that using PVP and SiO_2_ nanoparticles is supportive in driving the sensitivity at the evaluated frequencies. Different hydrophilic properties mean distinct ways to interact and react with water molecules. In particular, in Figure 5, SiO_2_ PVA:PVP_0%_ and SiO_2_ PVA:PVP_25%_ capacitors exhibit higher capacitance sensitivities than the PVA:PVP_0%_ capacitor, thereby revealing the positive impact of the charge pathways provided by the nanoparticles on the sensitivity.

Figure 5 also reveals that incorporating PVP and SiO_2_ into the PVA-ILGPE increases the capacitance sensitivity within the frequency range of 1 kHz to 100 kHz. These findings could be ascribed to the number of active sites per volume available in the sensing layer. Firstly, the presence of PVP in the sensing layer composition implies a relevant change in the available active sites per volume in the sensing layer because of its amorphous nature. Secondly, the presence of SiO_2_ nanoparticles affects the sensitivity since SiO_2_ fillers can increase the available surface area and number of active sites per volume [1]. This effect could arise from the reduction in the crystallinity of the ILGPEs, which is supported by the findings revealed in Figure 3, and the high area-to-volume ratio of the nanoparticles available for ion exchanges. Finally, it is noteworthy that the device that exhibits the highest capacitance sensitivities is the SiO_2_ PVA:PVP_25%_ capacitor, revealing the positive contribution of the combination of the SiO_2_ and PVP benefits. This result validates the hypothesis that incorporating SiO_2_ nanobeads and PVP can stimulate a reduction in the degree of crystallinity of PVA. Therefore, the resultant electrolyte exhibits a more amorphous layer with a rougher surface, increasing the sensing area and enhancing the sensitivities.

**Figure 6:**
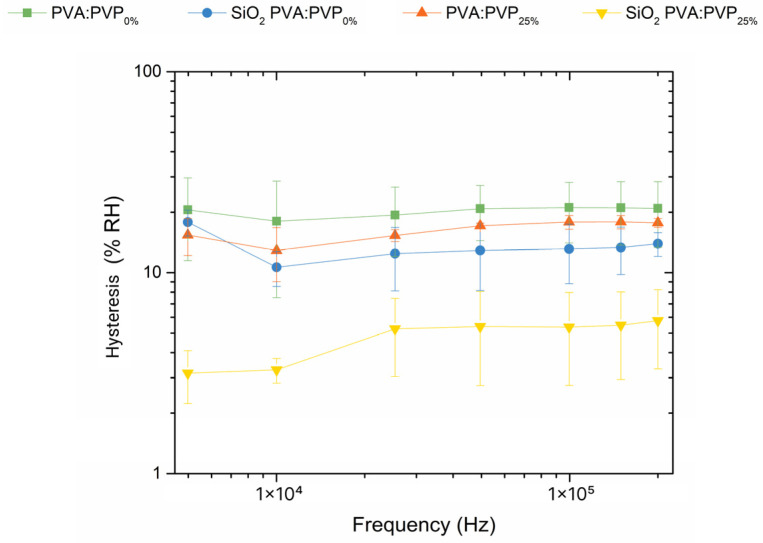
Comparison of the capacitance hysteresis values exhibited by PVA:PVP_0%_-ILGPE-, SiO_2_ PVA:PVP_0%_-CILGPE-, PVA:PVP_25%_-ILGPE-, and SiO_2_ PVA:PVP_25%_-CILGPE-based capacitors at different possible working frequencies.

In addition to the analysis of the impact of PVP and SiO_2_ nanoparticles on the sensitivity, the hysteresis parameter has also been analysed. Hysteresis refers to the highest deviation between the obtained ILGPE and SiO_2_ CILGPE capacitor responses during desorption and adsorption processes. Factors such as porous structures or surface morphology of the sensing layer mainly dominate the hysteresis parameter [42]. Therefore, hysteresis is a significant parameter in evaluating the sensing performance and understanding the absorption principle of the humidity sensor. Initially, to obtain the adsorption responses, the capacitors were exposed to a controlled environment in which the RH was gradually increased from 20 %RH to 90 %RH over a specific time. Afterwards, to measure the desorption responses, the relative humidity was progressively decreased from 90 %RH to 20 %RH. The impedance spectra of the ILGPE and SiO_2_ CILGPE capacitors were obtained at each relative humidity variation. After calculating the capacitance values at each specific %RH during the adsorption and desorption processes, the hysteresis value was calculated using the following Equation (4) [1,13]:

(4) $$H=\frac{max\;\left(\gamma_{x{\%}_{D}}-\gamma_{x{\%}_{A}}\right)}{S}$$

where $\gamma_{x{\%}_{D}}$ and $\gamma_{x{\%}_{A}}$ are the obtained capacitance values during desorption and adsorption at the steady humidity level, respectively, and S is the sensitivity. Figure 6 compares the hysteresis characteristics exhibited by the fabricated devices at the evaluated frequencies.

As is noticeable from Figure 6, SiO_2_ PVA:PVP_0%_ and SiO_2_ PVA:PVP_25%_ capacitors exhibit lower hysteresis values than their electrolyte counterparts without embedded SiO_2_ nanoparticles in the electrolyte. This finding indicates that SiO_2_ CILGPE capacitors are more susceptible to humidity changes than ILGPE-based capacitors, exposing the dominant role of silica nanoparticles in determining the hysteresis. SiO_2_ nanofillers could generate pathways that can facilitate the diffusion of water molecules since they indicate a connection between the different levels of the sensing layer, thereby promoting water molecule diffusion. Furthermore, it is essential to highlight the constructive contribution of PVP in SiO_2_ CILGPE capacitors. The simultaneous existence of SiO_2_ nanobeads and PVP in the PVA-based ILGPE capacitor exhibits the lowest hysteresis values in this study, revealing the favourable combination of these materials. The reduction in hysteresis may be primarily attributed to the amorphous nature of PVP. The inherent amorphous structure of PVP disrupts the crystallinity of the PVA matrix, increasing the amorphousness of the SiO_2_ CILGPE and leading to a reduction in zones with a crystalline phase that can hinder the diffusion of water molecules, thus facilitating their transport. Moreover, the hydrophilicity of PVP stimulates the formation of additional hydrogen bonds with water, affecting the retention and release of water molecules and increasing the susceptibility of SiO_2_ CILGPE to %RH variations. These findings are consistent with those of [16,42], which indicate that high surface-to-volume-ratio materials result in excellent humidity-sensing performance, although their intrinsic chemical and physical characteristics are more susceptible to changes, which directly influence the hysteresis.

**Figure 7:**
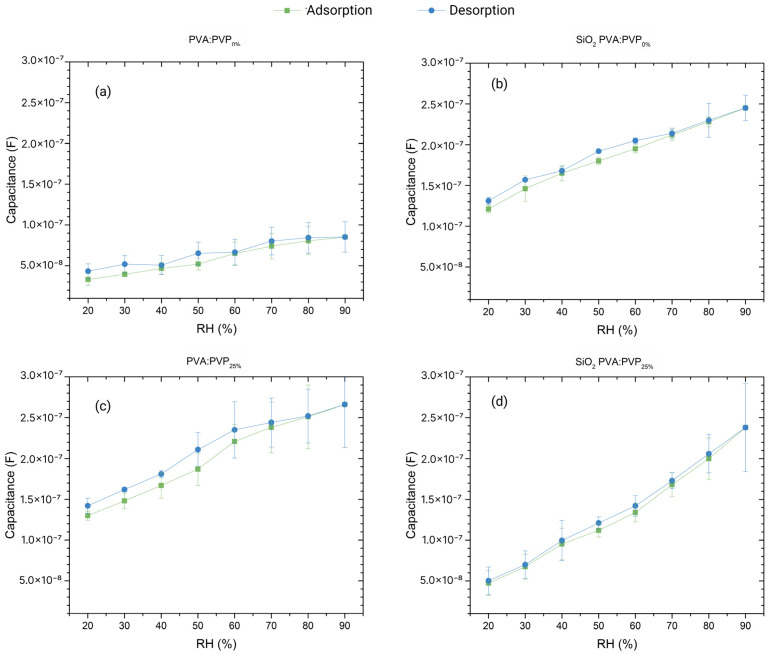
Comparison of the capacitance responses during the adsorption and desorption processes exhibited by (**a**) PVA:PVP0%-ILGPE-, (**b**) SiO_2_ PVA:PVP0%-CILGPE-, (**c**) PVA:PVP25%-ILGPE-, and (**d**) SiO_2_ PVA:PVP25%-CILGPE-based capacitors at their potential operating frequencies.

As a summary, Figure 7 depicts the capacitance responses of the PVA:PVP_0%_, SiO_2_ PVA:PVP_0%_, PVA:PVP_25%_, and SiO_2_ PVA:PVP_25%_ capacitors during the adsorption and desorption processes at the frequencies that exhibit higher sensitivities and lower hysteresis values. Consequently, for PVA:PVP_0%_ capacitors, the optimal working frequency is 50 kHz, while for SiO_2_ PVA:PVP_0%_, PVA:PVP_25%_, and SiO_2_ PVA:PVP_25%_ capacitors, it is 10 kHz. Notably, the PVA:PVP_0%_ ILGPE capacitor shows a capacitance response with a sensitivity of about 739 pF/%RH. As explained above, the incorporation of silicon dioxide nanoparticles stimulates increased sensibility and decreased hysteresis. As a result, the SiO_2_ PVA:PVP_0%_ CILGPE capacitor exhibits capacitance sensitivities of about 1780 pF/%RH with a hysteresis value of 10.64 %RH.

Furthermore, adding PVP also enhances the sensing performance, leading to capacitance sensitivities of 1950 pF/%RH with maximum hysteresis of about 12.89 %RH. Finally, as is noticeable, the SiO_2_ PVA:PVP_25%_-ILGPE-based capacitor shows the highest capacitance sensitivity with the lowest hysteresis values, exhibiting capacitance sensitivities of 2730 pF/%RH with the lowest maximum hysteresis about 3.28 %RH, respectively, thereby revealing the positive effect of combining PVP and SiO_2_ fillers. The lowest hysteresis value can be mainly ascribed to the presence of SiO_2_ nanoparticles. As explained earlier, SiO_2_ nanoparticles may facilitate the diffusion of water molecules by connecting different levels of the sensing layer (top and bottom). In addition to this effect, the presence of PVP, which can increase the amorphousness, could reduce the barriers to water molecule movement. Irregular arrangements may provide more pathways for water molecule diffusion, which can reduce the hysteresis.

On the other hand, regarding the higher sensitivity, the coexistence of PVP and SiO_2_ nanoparticles increases the amorphousness of the sensing layer. Therefore, their presence can increase the surface area where water molecules can be adsorbed. Additionally, as both materials are hydrophilic, their coexistence further increases the active sites available for the interaction between water molecules and the sensing layer, increasing the susceptibility to humidity changes.

Additionally, Table 1 provides an overview of the linearity performance exhibited by PVA:PVP_0%_, SiO_2_ PVA:PVP_0%_, PVA:PVP_25%_, and SiO_2_ PVA:PVP_25%_ capacitors at 10 kHz within the suggested detection range. The regression analysis conducted on ILGPE- and SiO_2_ CILGPE-based capacitors demonstrates the existence of a linear relationship between the relative humidity and capacitance responses. The resulting high R-squared values show a reliable and accurate linear fit, further emphasising the linear performance within the proposed sensing range. Furthermore, regarding the detection limit, the criteria for selecting sensing limits is based on discarding the relative humidity levels that reveal differences lower than 3% compared to their previous %RH levels. Consequently, the PVA:PVP_0%_-based capacitor is suggested to operate within the 20–70 %RH sensing range. From Table 1, it can be concluded that the addition of SiO_2_ and PVP could expand the humidity-sensing span, revealing the positive impact of these materials on the sensing range. Additionally, as the sensitivity can also be defined as the slope of the calibrated fitting response of a sensor [1], this table also shows the sensitivity values of the calibrated responses.

As a result of the findings discussed above, it can be concluded that the most suitable electrolyte for acting as a humidity-sensing layer is the SiO_2_ PVA:PVP_25%_ CILGPE in terms of sensitivity and hysteresis. Nevertheless, it is important to investigate the reliability of a sensor in the context of humidity sensing. To evaluate the reliability of the optimised SiO_2_ CILGPE, the long-term stability, the effect of the temperature on the capacitance response, and the impact of the cyclability on the hysteresis parameter have been investigated.

**Figure 8:**
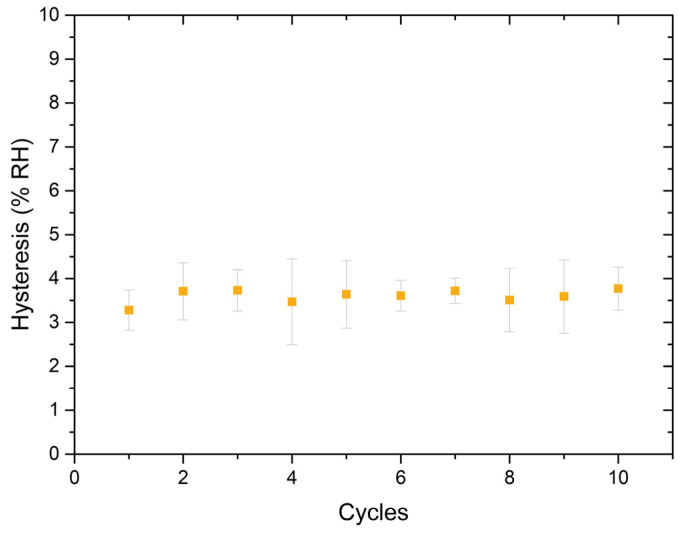
Exhibited evolution of the hysteresis parameter over ten cycles.

The cyclability of the optimised sensor has been examined since it is a crucial parameter in determining the reliability and durability of a sensor. Cyclability refers to the ability exhibited by a sensor to maintain its sensing performance over many cycles of usage. In particular, the cyclability has been analysed by measuring the adsorption and desorption responses for ten cycles to determine their impact on the hysteresis parameter. The study of the hysteresis parameter across several cycles provides a first insight into how consistent and reliable the capacitive humidity response can be over repeated cycles. Additionally, understanding how the cyclability affects the hysteresis can give information about the sensor’s durability and stability. Figure 8 shows the obtained hysteresis over ten consecutive cycles, providing insights into the stability and repeatability of the optimised sensor. In particular, Figure 8 illustrates that the capacitance response of a SiO_2_ PVA:PVP_25%_ capacitor exhibits similar hysteresis during ten cycles, showing hysteresis values around 3.6 %RH. This finding may indicate that, in terms of hysteresis, the SiO_2_ PVA:PVP_25%_ capacitor could exhibit a consistent and reliable humidity-sensing performance over ten cycles.

**Figure 9:**
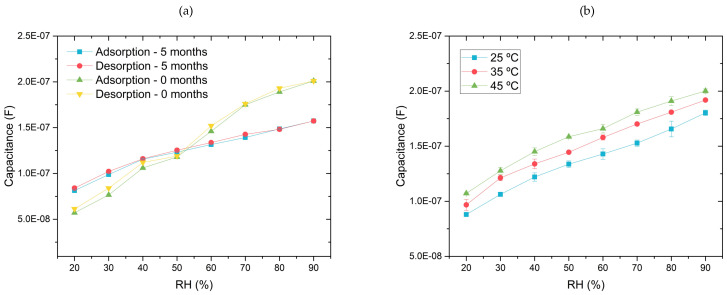
Comparison of (**a**) a capacitance response after five months and (**b**) capacitance responses of the SiO_2_ PVA:PVP_25%_ capacitor at 25 °C, 35 °C, and 45 °C.

Furthermore, to analyse the long-term stability, a SiO_2_ PVA:PVP_25%_-based capacitor was exposed to natural environmental conditions for five months after its fabrication. A comparison between the responses of the SiO_2_ PVA:PVP_25%_ five months after the fabrication process and in its initial state is displayed in Figure 9a. This analysis provides initial insights into the ability of the SiO_2_ PVA:PVP_25%_ CILGPE to maintain its sensitivity and hysteresis characteristics.

Figure 9a compares the initial and the five-month capacitance responses, exhibiting a humidity-sensing deterioration. After five months, the capacitance response shifts to higher values and displays a reduced sensitivity. Notably, the initial capacitance responses exhibit a calibrated sensitivity of 2140 pF/%RH, while five months later, the same device presents a reduction of about 50% in its sensitivity, thereby revealing a sensitivity of 1024 pF/%RH. However, despite this observed sensitivity degradation, the SiO_2_ PVA:PVP_25%_ CILGPE-based capacitors still display a linear behaviour, thereby indicating the possibility of compensation for this degradation. This alteration in the humidity capacitance response indicates some concerns that merit further investigation. Therefore, it is advisable to delve deeper through additional research aimed at studying and mitigating this degradation.

Considering that temperature can impact humidity sensors’ long-term stability and durability, a five-month-old device has also been employed in temperature-dependence analysis. This approach provides insights into SiO_2_ CILGPE-based capacitors’ performance and endurance under fluctuating thermal conditions, revealing more information about their reliability over time. As is widely known, most hygroscopic materials reveal variations in their characteristics under different temperatures, exhibiting a dependency on temperature. This effect is particularly noticeable in impedimetric and capacitance sensors. Therefore, the evaluation of how temperature affects humidity-sensing responses may result in the mitigation of its impact. Thus, the reliability of humidity sensors can be improved by compensating for the potential erroneous humidity readings. To determine the effect of the temperature variation on the humidity-sensing performance of the optimised sensing layer, IS measurements of RH at three different temperatures (25 °C, 35 °C, and 45 °C) have been performed at the selected working frequency. Figure 9b illustrates the resulting capacitive humidity responses at the proposed working frequency of 10 kHz under different temperatures. Notably, Figure 9b shows that the obtained capacitance responses of the optimised sensing layer are shifted to higher values with the increase in ambient temperature. This behaviour has been previously reported and indicates that an increase in temperature can provide additional energy to charge carriers, leading to a stronger thermal movement of the water molecules [13,43].

As a consequence, there is an increase in the charge carriers’ mobility, which can also increase the capacitance values. Furthermore, as can be noticed in Figure 9b, the obtained capacitance responses at different temperatures depict similar linear trends without exhibiting significant sensitivity variations. This finding indicates a suitable temperature dependence to function as a humidity sensor since the deviation change promoted by the temperature increase can be easily compensated.

**Figure 10:**
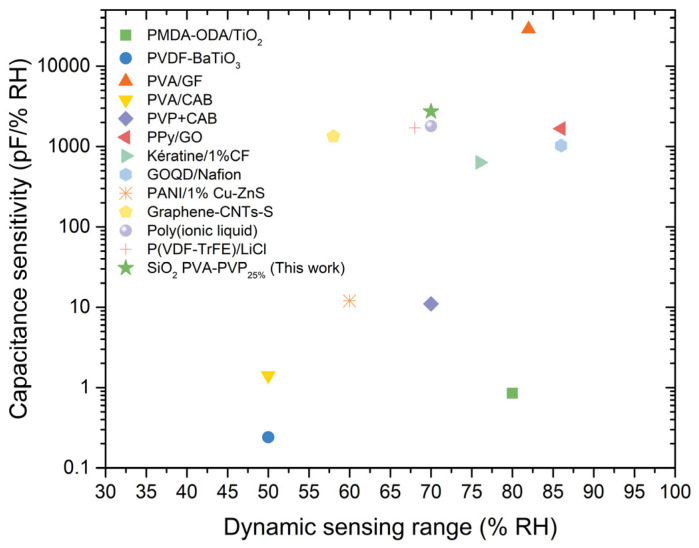
Comparison of the sensing parameters exhibited by some reported humidity sensors in SOA and the SiO_2_ PVA:PVP_25%_ capacitor.

Finally, Figure 10 and Table 2 compare the SiO_2_ PVA:PVP_25%_ capacitors’ sensing properties with those previously exhibited in the SOA. Figure 10 shows that, when compared with some recently reported capacitive humidity sensors based on pyromellitic dianhydride (PMDA)-oxydianiline (ODA)/TiO_2_ [13], polyaniline (PANI)/1% Cu–ZnS [33], poly(ionic liquid) [44], PPy/graphene oxide (GO) [9], graphene oxide quantum dots (GOQD)/Nafion [11], PVA/cellulose acetate butyrate (CAB) [6], keratine/1% carbon fibres (CF) [10], graphene–carbon nanotube (CNT)–silicone adhesive (SA) [34], PVDF-BaTiO_3_ [15], GO-doped poly(vinylidene fluoride-trifluoroethylene) (P(VDF-TrFE))/lithium chloride (LiCl) [41], or PVP+CAB [8], the SiO_2_ PVA:PVP_25%_-CILGPE capacitor has relevant detection parameters. These prior studies combine nanofillers or salts with polymers or use ILs to enhance sensitivity and hysteresis in humidity sensors. For instance, incorporating carbon fibres into a keratin matrix resulted in a capacitance sensitivity of 633.12 pF/%RH across a wide sensing range. Similarly, the PPy and graphene oxide composite yielded a low-hysteresis humidity sensor with a sensitivity of 1670.3 pF/%RH. Furthermore, the polymerisation of an ionic liquid or adding salt into a PVA matrix to create microstructures revealed capacitance sensitivities of 1.8 nF/%RH with low hysteresis and 1.41 pF/%RH, respectively.

In contrast, this study explores the impact of silica nanoparticles, PVP, and their combination on an ILGPE, revealing the beneficial effects of the simultaneous presence of these materials and exhibiting a higher calibrated capacitance sensitivity with a value of 2660 pF/%RH and low hysteresis. Therefore, this work introduces a novel composite that blends polymers, nanofillers, and an ionic liquid. The resulting composite is based on eco-friendly materials and uses an easy and fast fabrication process. This approach exhibits an enhancement of capacitance sensitivity and hysteresis. Finally, as could be concluded from Table 2 and Figure 10, the SiO_2_ PVA:PVP_25%_-CILGPE-based device outperforms some reported capacitive humidity sensors in terms of sensitivity. Approximately 64% of the studies summarised in the table reported capacitance sensitivities below 1500 pF/%RH. The good sensitivity and hysteresis of the SiO_2_ PVA:PVP_25%_-CILGPE-based capacitor, combined with its simple and cost-effective fabrication process and possible easy compensation of its temperature dependence, position the optimised device as a viable option for a humidity sensor.

### 2.4. Humidity-Sensing Mechanism

Previous research has established a link between the electrical and physical behaviours of humidity sensors by fitting their complex impedance spectroscopy data using equivalent circuits, allowing for the investigation of the sensing mechanism [9,16,43]. Consequently, to investigate the working mechanism of the PVA:PVP_0%_, SiO_2_ PVA:PVP_0%_, and PVA:PVP_25%_ capacitors and the impact of the addition of SiO_2_ nanoparticles, PVP, or both on the sensing mechanism, the variation in the values of the elements that compose the equivalent circuit have been analysed as a function of %RH. As previously explained, the obtained impedance spectra can be modelled using an equivalent circuit. This electrical circuit consists of a resistance (*R_s_*) in series with the parallel combination of a *CPE* and a leakage resistance (*R_p_*). The *R_s_* parameter corresponds to the bulk resistance of the electrolyte and the electrodes, while the *CPE* is attributed to the double-layer capacitances. Alterations in humidity mainly affect the *CPE*, which is noticeable in the *Q_CPE_* parameter and the value of *R_s_*. Consequently, the impact of moisture on both parameters has been analysed, as any changes in the electrical characteristics of the electrolyte are reflected in these parameters.

**Figure 11:**
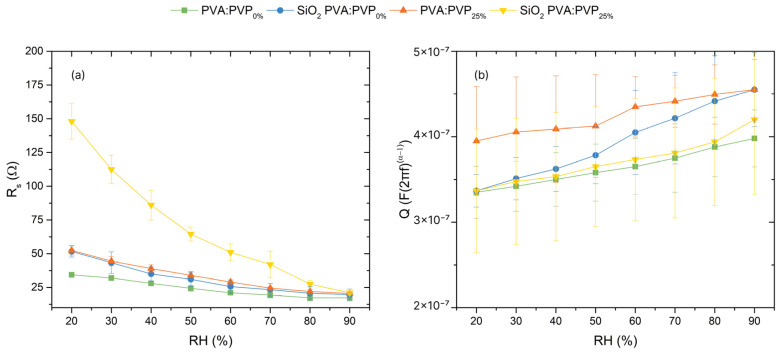
Trends in the (**a**) *R_s_* (series resistance) and (**b**) *Q_CPE_* (ideal admittance of the *CPE*) values at different relative humidity levels.

On the one hand, Figure 11 shows the evident tendency followed by the values of *R_s_* and *Q_CPE_* with changes in humidity. Notably, Figure 11a illustrates the obtained *R_s_* values for PVA:PVP_0%_, SiO_2_ PVA:PVP_0%_, PVA:PVP_25%_, and SiO_2_ PVA:PVP_25%_ capacitors across different %RH levels, exhibiting their respective trends. As displayed in Figure 11a, there is an evident reduction in *R_s_* values as humidity increases. This finding aligns with the impedance modulus trend and indicates an enhancement in the charge conduction that reduces the electrolyte resistance. This improved charge conduction could be the result of an increase in the charge content and enhanced ionic mobility. The higher ion density can be attributed to the ability of PVA, PVP, and SiO_2_ nanoparticles to absorb and interact with water molecules from the environment. This effect is also noticeable in the behaviour of the *Q_CPE_* parameter relative to relative humidity levels. Figure 11b shows that *Q_CPE_* values increase with rising humidity. This behaviour is widely exhibited by capacitive humidity sensors [16,33,34]. The higher *Q_CPE_* values due to the %RH increase also indicate an enhancement in charge density, thereby promoting the formation and enhancement of electrical double-layer capacitance.

**Figure 12:**
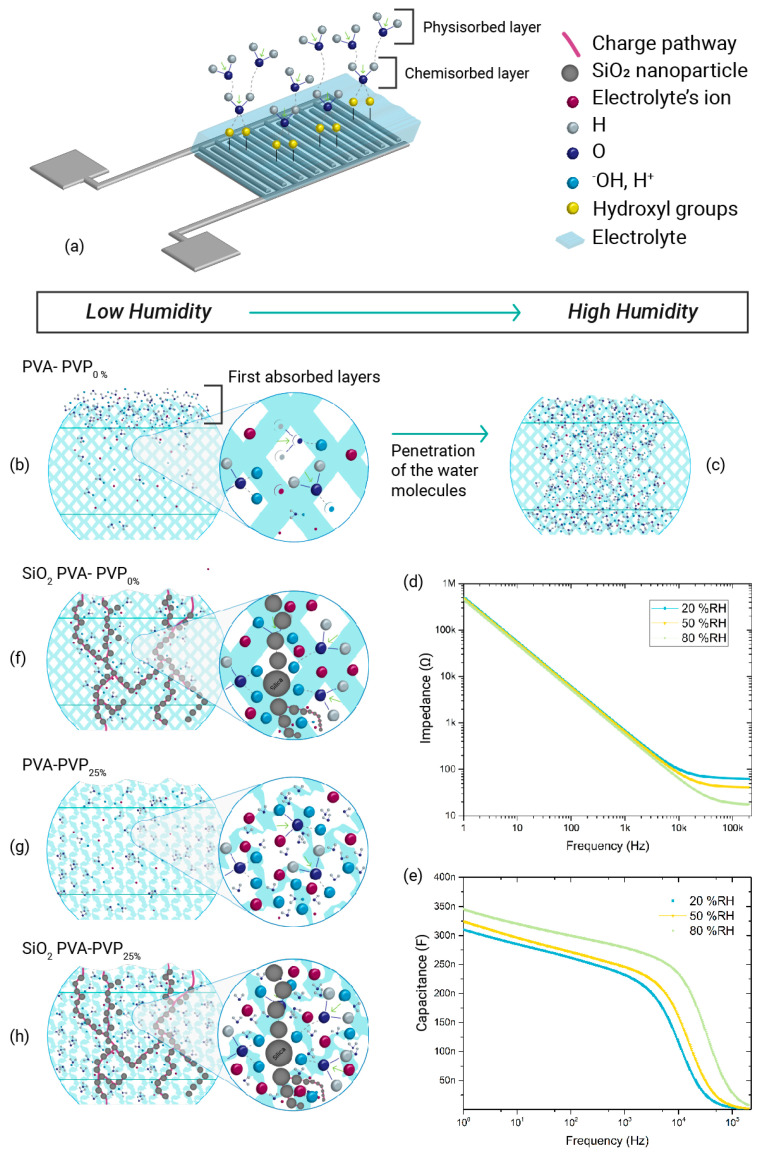
Proposed operation principle of the fabricated ILGPE and SiO_2_ CILGPE sensors. (**a**) Graphical representation of a fabricated ILGPE sensor and the first chemisorbed and physisorbed water layers. (**b**) longitudinal section of PVA:PVP0% ILGPEs at low humidity levels. (**c**) longitudinal section of PVA:PVP0% ILGPEs at high humidity levels, illustrating the penetration of water molecules with the increase in humidity. (**d**) comparison of the impedance vs. frequency curves at different frequencies. Higher humidity levels imply lower impedance values. (**e**) comparison of the capacitance vs. fre-quency curves at different frequencies. Higher humidity levels result in higher capacitance values. (**f**) longitudinal section of SiO_2_ PVA:PVP0% CILGPEs. The grey spheres, connecting the top and bottom of the sensing layer, represent the SiO_2_ nanoparticle pathways that can facilitate the diffu-sion of water molecules. (**g**) longitudinal section of PVA:PVP25% ILGPEs. The addition of PVP dis-rupts the crystallinity of PVA, increasing the amorphousness and, hence, the available active sites to interact with water molecules. (**h**) longitudinal section of SiO_2_ PVA:PVP25% CILGPEs. This type of CILGPE combines the benefits of incorporating silica nanoparticles and PVP.

Therefore, the humidity-sensing mechanism works as depicted in Figure 12. Initially, at low %RH levels, an initial interaction transpires between the surface of the sensing layer and the water molecules present in the environment, leading to some water molecules being trapped at the outermost levels of the sensing layer (Figure 12a,b). This first interaction forms a chemisorbed layer formed by hydrogen bonds between water molecules and the hydroxyl groups in the sensing material, as explained in [1,20,45]. This interaction is mainly attributed to the inherent hydrophilicity of the sensing layer, which has a minor impact on the charge conduction since chemisorbed molecules are not free to move [1]. As the humidity rises, the sensing layer transitions from chemisorption to physisorption interactions. At this point, the physically adsorbed water molecule layers add to the chemically adsorbed layer, forming a continuous water molecule layer. These additional water molecules start to contribute to the charge conduction of the sensing layer due to the conduction of some extra ions [20,45]. This process leads to a decrease in the *R_s_* parameter and an increase in the *Q_CPE_* values. With further increases in the humidity, a fraction of the confined water molecules on the most superficial levels start to penetrate the polymeric layer, revealing the existence of an internal–external density balance of water molecules until a state of equilibrium is reached (Figure 12c). At this point, the electrical behaviour is mainly controlled by charge conduction, resulting in even lower *R_s_* and higher *Q_CPE_* values. This finding is attributed to improved charge conduction due to (i) enhanced charge mobility, as the presence of continuous water molecules may allow higher mobility of the ions in the sensing layer, and (ii) a higher effective charge density, owing to the extra ions (H_3_O^+^, H^−^) contributing to the conduction. Consequently, higher relative humidity levels imply an increase in charge density inside the sensing layer, reducing the impedance of the polymer electrolyte and boosting capacitance values (Figure 12d,e).

In contrast, during a decrease in %RH, this internal–external density balance is disrupted, and the trapped water molecules inside the sensing layer try to escape adsorption and initiate diffusion. This diffusion process occurs gradually, commencing with freeing the trapped water molecules at the outermost levels. Subsequently, the trapped molecules at the deeper layers start to ascend and diffuse, attaining equilibrium, decreasing the number of charges precedent from the water molecules’ interactions, increasing the impedance, decreasing the capacitance, and, hence, increasing the *R_s_* and decreasing the *Q_CPE_* values.

This proposed sensing mechanism is consistent with those discussed in [10,16,20,33,40,42,45]. These previous studies explain that the humidity-sensing mechanism of polymeric sensors is governed by the interaction between the humidity-sensitive layer and water molecules, which is mainly explained by chemisorption and physisorption processes, as well as Grotthus chain reactions. The hydrophilic polymer possesses active sites that facilitate the interaction with water molecules, promoting an increase in the number of charge carriers within the host polymer.

On the other hand, Figure 11a,b also illustrate the impact of the addition of SiO_2_, PVP, and both materials to the PVA-based ILGPE on the sensing mechanism. Firstly, Figure 11a displays how the composition of the sensing layer affects the *R_s_* behaviour at different %RH levels. In particular, as %RH increases from 20 to 90%, the *R_s_* values for PVA:PVP_0%_, SiO_2_ PVA:PVP_0%_, PVA:PVP_25%_, and SiO_2_ PVA:PVP_25%_ capacitors ranged from 34 Ω, 51 Ω, 52 Ω, and 148 Ω to 17 Ω, 19 Ω, 20 Ω, and 21 Ω, respectively. Notably, incorporating PVP and SiO_2_ fillers leads to higher *R_s_* values. Higher *R_s_* values could suggest a hindrance to charge transport at lower humidity levels, indicating that charge carriers require more energy to be transported within the polymer electrolyte [12]. This effect is most pronounced at low humidity levels, where PVP and SiO_2_ nanoparticles may attempt to retain the charge due to their hydrophilic properties, impeding the mobility of some charge carriers.

Secondly, Figure 11b shows the evolution of the *CPE* element with the humidity, depending on the composition of the electrolyte, through the *Q_CPE_* parameter. At 20 %RH and 90 %RH, the PVA:PVP_0%_, SiO_2_ PVA:PVP_0%_, PVA:PVP_25%_, and SiO_2_ PVA:PVP_25%_ capacitors exhibit *Q_CPE_* values range from 0.32 μF, 0.34 μF, 0.39 μF, and 0.34 μF to 0.39 μF, 0.45 μF, 0.45 μF, and 0.42 μF, respectively. This trend aligns with the abovementioned capacitance responses. The higher *Q_CPE_* values may be ascribed to the amorphousness caused by adding PVP and SiO_2_ nanoparticles. Additionally, the higher variations in *Q_CPE_* values with increasing humidity can also be attributed to the expanded air-contact area on the surface of the sensing layer, enhancing the sensitivity of the devices. This hypothesis is consistent with the results from the surface topography analysis and the observed capacitance responses.

In summary, the results exhibit that adding PVP to a PVA-based gel polymer electrolyte leads to higher capacitance values in response to humidity variation (Figure 11b). This effect could be ascribed to PVP’s amorphous nature and hydrophilic properties. Firstly, the excellent miscibility between PVP and PVA due to hydrogen bonding formation, coupled with the amorphousness of PVP, decreases the degree of crystallinity of the PVA matrix [37,38,39]. The reduction in the crystalline phases and increase in the amorphous phase enhance the ionic conductivity of the ILGPE [36], which can stimulate a capacitance Increase. Secondly, the amorphousness induced by PVP increases the number of available active sites per volume in the sensing layer. This, combined with the hydrophilic nature of PVP, may enhance the interaction between the sensing layer and the water molecules, leading to a higher charge density within the electrolyte layer and, hence, higher variations in the capacitance (Figure 12g,h).

Moreover, SiO_2_ CILGPE capacitors show more significant changes in *Q_CPE_* values than ILGPE-based capacitors. This effect can be mainly attributed to the active sites for ion exchange provided by SiO_2_, which modifies the interaction of charge carriers with the sensing layer. As revealed in [20], embedding SiO_2_ nanoparticles into a polymer matrix offers several beneficial effects, including an increased charge contribution owing to their hydrophilic nature and the creation of new effective charge transport pathways, among others. These effects provided by the presence of SiO_2_ nanoparticles can contribute to the overall sensing mechanism. Incorporating nanoparticles in the hydrophilic polymer matrix enhances the available surface area for water interaction, increasing the amount of water molecules adhered to the sensing layer. This is achieved by introducing active sites that stimulate water adsorption [1,16], leading to improved binding of water molecules. Consequently, the probability of capturing water molecules increases, resulting in an augmented charge density within the humidity-sensitive layer.

Furthermore, as depicted in Figure 12f,h, the addition of silicon dioxide nanoparticles can introduce nanostructures that establish connections between different levels of the sensing layer. These silica structures can act as pathways for charge carriers and water molecules, facilitating their movement through the sensing layer. Consequently, these nanostructures can play a crucial role during desorption, providing a path for water molecules to traverse between the bottom and surface of the humidity-detection layer, assisting in the diffusion of water molecules into the environment and enhancing the water molecules’ equilibrium. This structural effect can improve the responsiveness of the humidity sensing, leading to a reduction in the hysteresis parameter. This hypothesis is consistent with the results shown in the analysis of the hysteresis parameter.

## 3. Conclusions

In this work, different ionic liquid gel polymer electrolytes (ILGPEs) consisting of CH3COONH4, BmImBr, SiO_2_ nanoparticles, and different concentrations of PVA and PVP have been fabricated and drop-casted over interdigitated aluminium electrodes to fabricate ILGPE-based and SiO_2_ CILGPE-based capacitors. The capacitors have been characterised via the IS method in the humidity range of 20–90 %RH and modelled using a Cole–Cole model as an equivalent circuit. The results show that using PVP and SiO_2_ nanoparticles enhances the sensitivity. Notably, SiO_2_ CILGPE capacitors exhibit higher sensitivity and lower hysteresis values than their counterparts due to (i) an increased air-contact sensing area, (ii) an increase in the number of active sites per volume available in the sensing layer stimulated by the disruption of the crystalline structure of PVA and the hydroxyl groups in the nanoparticles, and (iii) additional charge transportation pathways, which boost the ion conduction and facilitating the water molecule diffusion, therefore revealing the dominant role of silica in the hysteresis. Furthermore, the coexistence of PVP and silica nanoparticles further enhances the hysteresis and sensitivity by combining their hydrophilicity and ability to increase the amorphousness of the PVA:PVP_0%_ ILGPE. This amalgamation provides additional active sites for water interaction and pathways, further increasing the interaction with water molecules and facilitating their diffusion.

Consequently, the results suggest that the SiO_2_ PVA:PVP_25%_ CILGPE is the optimal electrolyte, exhibiting calibrated capacitance sensitivities around 2660 pF/%RH. These findings, coupled with its easy and fast fabrication process and environmentally friendly materials, outperform some reported capacitive humidity sensors in terms of sensitivity without compromising the hysteresis. Nevertheless, future research could investigate the optimisation of nanoparticle concentrations or the use of hydrophilic porous fillers, such as HNTs, to further improve the sensing properties of the optimal humidity-sensitive electrolyte.

## 4. Materials and Methods

### 4.1. Fabrication Process Steps

**Figure 13:**
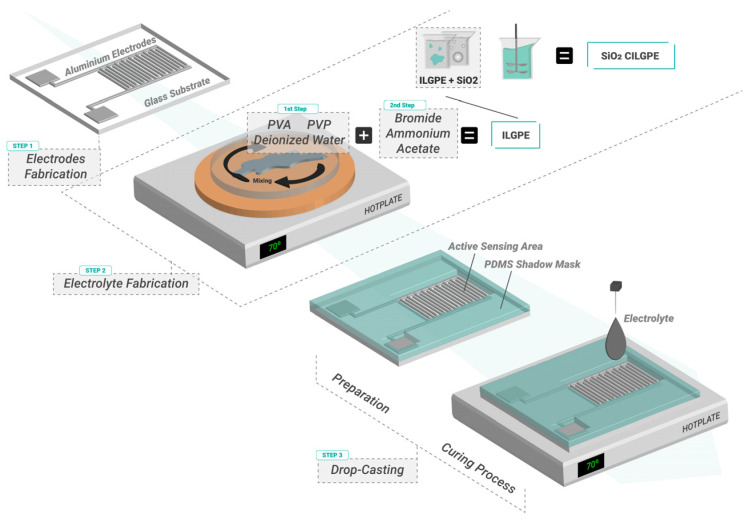
Fabrication method to manufacture ILGPE and CILGPE capacitors. Step 1: fabrication of aluminium interdigitated electrodes. Step 2: electrolyte fabrication. Step 3: electrolyte deposition by drop casting and curing at 70 °C.

Figure 13 illustrates the fabrication process employed to produce the ILGPE and SiO_2_ CILGPE capacitors. The fabrication process initiates with the creation of interdigitated aluminium electrodes with a total electrode area of 0.1962 cm^2^ (step 1). The fabricated electrolytes are deposited onto the electrodes by a drop-casting method. The first ionic liquid gel polymer electrolyte is based on the composition shown in [32,46]. Its formulation contains polyvinyl alcohol (PVA), ammonium acetate (CH₃COONH₄), and 1-butyl-3-methylimidazolium bromide (BmImBr). The second ILGPE is also composed of polyvinylpyrrolidone (PVP), CH₃COONH₄, and BmImBr. Furthermore, monodisperse SiO_2_ nanoparticles in a colloidal suspension are employed to create composite ILGPEs. The PVA and PVP serve as host polymers, ammonium acetate functions as salt and plasticiser, 1-butyl-3-methylimidazolium bromide acts as the ionic liquid, and silica nanobeads act as doping fillers.

The preparation of the PVA:PVP_0%_ and SiO_2_ PVA:PVP_0%_ electrolytes follows the fabrication processes exhibited in [32,46], respectively. As shown in step 2 of Figure 10, the preparation method of ILGPEs and SiO_2_ CILGPEs starts by dissolving the host polymer (Hp) in 10 mL of deionised water, followed by stirring the mixture for 10 min at 70 °C. Immediately, a blend of 420 mg of CH₃COONH₄ in a weight ratio of 70:30 for Hp:CH₃COONH₄ is added, and 4.24 g of 1-butyl-3-methylimidazolium bromide is incorporated into the Hp–CH₃COONH₄ mixture. The final blend is manually stirred and heated at 70 °C for 15 min. Afterwards, for the SiO_2_ CILGPEs, a volume ratio of 50:50 is used for the Hp–CH₃COONH₄–BmImBr–H₂O:SiO_2_ nanobeads blend. In particular, 1 mL of SiO_2_ nanoparticles is mixed with the Hp–CH₃COONH₄–BmImBr aqueous mixture using an ultrasound bath for 10 min. Finally, as illustrated in the third step of Figure 10, the resulting gel polymer electrolytes are deposited onto the active electrode area using drop casting and subsequently cured at 70 °C for 35 min and 15 min, respectively. The resultant gel polymer electrolyte layers present a thickness between 300 and 600 μm and a length and width of 0.4 mm and 1 mm, respectively. Table 3 summarises the codification of the developed electrolyte-based capacitors and the composition of the electrolytes that compose them.

### 4.2. Methodology

The electrical performance of the developed ILGPE- and SiO_2_ CILGPE-based capacitors was measured using a HIOKI IM3590 (HIOKI, Dallas, TX, USA) impedance analyser. The IS measurements have been performed in the frequency range from 0.1 Hz to 200 kHz, applying a sinusoidal signal with an amplitude of 50 mV and without bias voltage.

In particular, the IS responses of three samples from different batches of PVA:PVP_0%_-ILGPE-, SiO_2_ PVA:PVP_0%_-CILGPE-, PVA:PVP_25%_-ILGPE-, and SiO_2_ PVA:PVP_25%_-CILGPE-based capacitors have been fabricated by the same abovementioned method and measured. The measurement setup and process follow similar configurations to those exhibited in [13]. First, the resultant capacitors were connected to a measurement board and placed inside a commercial environmental chamber at 25 °C. Coaxial cables connected the impedance analyser and the measurement board. The chamber’s humidity was increased from 20 %RH to 90 %RH to obtain the adsorption responses. On the contrary, to obtain the desorption responses, the relative humidity was decreased to 20 %RH in steps of 10 %RH.

The IS measurements obtained from PVA:PVP_0%_, SiO_2_ PVA:PVP_0%_, PVA:PVP_25%_, and SiO_2_ PVA:PVP_25%_ capacitors at different relative humidity levels have been fitted to show how the humidity affects the impedance characteristics of the devices and their potential as humidity sensors.

## Figures and Tables

**Table 1:** Comparison of the regression models, R-squared, and calibrated sensitivities obtained during the fitting of the capacitance responses of the PVA:PVP_0%_-ILGPE-, SiO_2_ PVA:PVP_0%_-CILGPE-, PVA:PVP_25%_-ILGPE-, and SiO_2_ PVA:PVP_25%_-CILGPE-based capacitors and their preferred sensing range at 10 kHz.

Identifier	Sensing Range (%RH)	Capacitance Response		
		Fitting Function	R^2^	Calibrated Sensitivity (nF/%RH)
PVA:PVP_0%_	20–70	0.79 × 10^−9^x + 0.16 × 10^−7^	0.989	0.79
SiO_2_ PVA:PVP_0%_	20–90	1.71 × 10^−9^x + 9.26 × 10^−8^	0.994	1.71
PVA:PVP_25%_	20–90	2.04 × 10^−9^x + 8.87 × 10^−8^	0.988	2.04
SiO_2_ PVA:PVP_25%_	20–90	2.66 × 10^−9^x − 1.38 × 10^−8^	0.985	2.66

**Table 2:** Comparison of sensitivity, hysteresis, sensing ranges, and fabrication methods of capacitive-type humidity sensors in the literature and the SiO_2_ PVA:PVP_25%_-CILGPE-based capacitor. TW (this work).

Sensing Material	Fabrication Method	Sensing Range (% RH)	Sensitivity (pF/% RH)	Hysteresis (% RH)	Ref.
PMDA-ODA/TiO_2_	Spin Coating	10–90	0.85	0.95	[13]
PVDF-BaTiO_3_	Spin Coating	40–90	0.2416	2.5	[15]
Mesoporous silica-PEDOT	-	11–95	~120	6	[16]
PVA/GF	Spin + Spray Coating	40–90	29,000	low	[5]
PVA/CAB	-	10–90	1.41	-	[6]
PVP+CAB	Inkjet Printing + Doctor Blade	20–80	11.4	-	[8]
PPy/GO	Drop Casting	11–97	1670.3	1.12	[9]
Keratine/1% CF	Drop Casting	16–92	633.12	1.33	[10]
Nafion/GOQD	Drop Casting	11.3–97.3	1025.97	4	[11]
PANI/ 1% Cu–ZnS	Spin Coating	30–90	12	1.56	[33]
Graphene–CNT–SA	Drop Casting + Doctor Blade	36–94	1336.7 −84.5 Ω/%RH	- -	[34]
Poly(ionic liquid)	Bar Printing	10–80	1800	Low	[44]
GO-doped P(VDF-TrFE)/LiCl	Drop Casting	25–93	1708.8	-	[41]
SiO_2_ PVA:PVP_25%_	Drop Casting	20–90	2660	3.28	TW

**Table 3:** Weight and volume ratios of the incorporated materials in the fabricated gel polymer electrolytes. The host polymer matrix is mixed with CH₃COONH₄ in a weight ratio of 70:30. BmImBr is added to the resulting mixture using a weight ratio of 75:25.

Identifier	Weight Ratio of Host Polymer (Hp) PVA:PVP	Volume Ratio of Hp–CH₃COONH₄–BmImBr–H₂O:SiO_2_ Nanoparticles
PVA:PVP_0%_	100:0	100:0
SiO_2_ PVA:PVP_0%_		50:50
PVA:PVP_25%_	75:25	100:0
SiO_2_ PVA:PVP_25%_		50:50

## Data Availability

All data and materials are available on request from the corresponding author. The data are not publicly available due to ongoing research using a part of the data.
